# The Salicyl-Sulphonic Acid Test for Albumen

**Published:** 1904-04-30

**Authors:** 


### THE SALICYL-SULPHONIC ACID TEST FOR ALBUMEN.

This test is performed by adding a few drops of a saturated aqueous solution of salicyl-sulphonic acid (25 grams of the crystals in 10 c.cm. of water) to 20 or 30 minims of urine in a very small test tube. If no precipitate occurs there is no proteid present. Should a precipitate occur the fluid is boiled. This causes the precipitate, if albuminous, to become coagulated and flaky, whereas proteoses in such circumstances clear up, but reappear as the fluid cools. Dr. Charles Murray,[1] of the University of Aberdeen, after an extended experience, is convinced of the safety, convenience, and delicacy of this test. No substance in the urine, apart from those of the proteid class, has been found to give the reaction. The same observer has investigated the relationship between the reaction of the urine and the precipitation of albumen in the ordinary test by boiling. His experiments show that 10 c.cm. of an albuminous urine to which one or two drops of nitric acid have been added may fail to give a precipitate when boiled; that the same result may follow when the amount of acid added is as much as 30 or 40 drops, whereas an intermediate quantity of acid, say 10 to 12 drops, will, when the urine is boiled, be followed by a considerable precipitate. The explanation of these results is that a very small quantity of acid causes the formation of acid albumen, which in a weak acid solution is not coagulable by heat; it is, however, coagulable in the presence of a certain proportion of mineral acid and hence the precipitate on boiling when not 1 or 2, but 10 or 12 drops of nitric acid have been used. When excess of acid is added a destructive influence exercised on the proteid may prevent precipitation on boiling. All this goes to show that the acidulation and boiling test as commonly practised has ready opportunities for erroneous conclusions. There is less chance of error if acetic acid is used instead of nitric, but even here a certain delicate degree of acidulation is necessary if absolutely accurate results are to be secured. In the salicyl-sulphonic acid test there is no danger of over-acidulation as even considerable excess does not dissolve the precipitated proteid. There is the further advantage that the acid is stable and non-caustic. Hence the crystals can be readily carried and the test applied at the bedside. All that is necessary is to add a little of the acid to half a drachm or so of the urine in a small test tube, and if a precipitate appears to boil the fluid. If the precipitate does not disappear, but, on the other hand, becomes coagulated and flaky, the conclusion that albumen is present is an absolute one. The non-appearance of a precipitate when the acid is added is equally a proof of the absence of albumen.

[1] Brit. Med. Jour., April 16, 1904.

